# Catechin-7-*O*-α-L-rhamnopyranoside can reduce α-MSH-induced melanogenesis in B16F10 melanoma cells through competitive inhibition of tyrosinase

**DOI:** 10.7150/ijms.72241

**Published:** 2022-06-27

**Authors:** Taehyeok Hwang, Hyo Jung Lee, Woo Sung Park, Dong-Min Kang, Mi-Jeong Ahn, Hyonok Yoon, Jae Cheal Yoo, Dong Kyu Moon, Dong Kyun Woo

**Affiliations:** 1 College of Pharmacy and Research Institute of Pharmaceutical Sciences, Gyeongsang National University, Jinju 52828, Republic of Korea.; 2 Department of Orthopedic Surgery and Institute of Health Sciences, School of Medicine and Hospital, Gyeongsang National University, Jinju 52727, Korea Republic of Korea.; 3 Axigen Co. Ltd., Seoul, Republic of Korea.

**Keywords:** Anti-melanogenic agent, Catechin-7-*O*-α-L-rhamnopyranoside, Melanogenesis, Skin aging, Tyrosinase, α-MSH

## Abstract

Although melanogenesis is a defense mechanism against ultraviolet (UV)-induced skin damage, abnormally excessive melanin production causes pigmentation disorders. Tyrosinase, as a key factor for melanin synthesis, plays an important role in inducing skin pigmentation. Therefore, the inhibition of tyrosinase is crucial in preventing skin pigmentation in the cosmetics and medicine fields. However, the majority of well-known tyrosinase inhibitors have been discontinued due to toxic effects on the skin or lack of selectivity and/or stability. In this study, we evaluated possible anti-melanogenic effects of catechin-7-*O*-α-L-rhamnopyranoside (C7R) isolated from the stem bark of *Ulmus parvifolia*, to discover a new tyrosinase inhibitor that has both safety and stability. When C7R was pretreated in B16F10 melanoma cells stimulated by α-melanocyte-stimulating hormone, this compound reduced melanin accumulation and murine tyrosinase activity. In line with these results, C7R inhibits tyrosinase purified from a mushroom *in vitro* like kojic acid and arbutin. Furthermore, C7R exhibited a competitive inhibition on a Lineweaver-Burk plot. Next, the underlying mechanisms of the C7R-mediated tyrosinase inhibitory effect were sought through docking simulation and pharmacophore analysis between tyrosinase residues and C7R. The results of these analyses showed that C7R had binding energy of -14.5kcal/mol, and indicated that C7R interacts with tyrosinase through an aromatic ring and various hydrophobic and hydrogen bonds. Together, our results suggest that C7R can be applied as a novel natural anti-melanogenic agent that inhibits tyrosinase.

## Introduction

Melanin is an essential factor in determining skin color and melanogenesis is a defense mechanism against ultraviolet (UV)-induced skin injury. Therefore, dark-skinned people have a lower risk of skin cancer than white-skinned people because the protective effect of melanogenesis by UV is higher 1. However, when melanin is uncontrolled and abnormal, it causes hyperpigmentation and pigmentation disorders such as freckles, age spots, and melasma due to excessive accumulation of melanin 2, 3. Generally, when the skin epidermis is exposed to UV, melanin is synthesized in melanocytes, leading to the making of melanosomes, and then the produced melanosomes are transferred to keratinocytes via dendrite 4-6.

When UVB is irradiated to the epidermis, melanogenesis is induced by several factors, of which alpha-melanocyte-stimulating hormone (α-MSH) triggers the melanin production. Mechanisms underlying melanosome transfer have also been studied a lot 7. α-MSH binds melanocortin 1 receptor to express microphthalmia-associated transcription factor (MITF), a transcription factor that expresses tyrosinase. When MITF gets phosphorylated, it moves into the nucleus to express tyrosinase 7. Tyrosinase is a key enzyme in the synthesis of melanin. It is involved in two catalytic reactions. First, through hydroxylation reactions, tyrosine is converted to 3,4-dihydroxy-L-phenylalanine (L-DOPA) by monophenolase activity of tyrosinase. Second, through oxidation reactions, L-DOPA is converted to DOPA-quinone by diphenolase activity of tyrosinase 8, 9.

As whitening compounds, many tyrosinase inhibitors including hydroxyquinone, koiic acid and arbutin, are studied in the pharmaceutical and cosmetic industries, but conventional agents often show low efficacy as well as side effects. Thus, many conventional compounds were initially used and then discontinued due to safety concerns 10-12. Therefore, it is important to search for a new compound that has a better effect on tyrosinase inhibitory activity and fewer side effects.

A deciduous arboreous tree, *Ulmus parvifolia* is distributed throughout the temperature zone of the Northern hemisphere, including China, Korea and Japan. The stem or root bark has been traditionally used for inflammation and gastric diseases 13. Recent studies have shown antioxidant, anti-inflammatory, and skin wound healing effects of *Ulmus parvifolia* 14, 15. Catechin-7-*O*-α-L-rhamnopyranoside (C7R), a catechin glycoside has been isolated as a major component from the stem bark of *Ulmus parvifolia*. Interestingly, catechin is known to inhibit melanin synthesis in B16F10 melanoma cells and tyrosinase expression 16. Thus, this study investigated a possible inhibitory effect of a catechin glycoside, C7R on skin melanogenesis.

## Results

### Inhibition of α-MSH-induced melanogenesis by C7R in B16F10 cell

**Figure 1:**
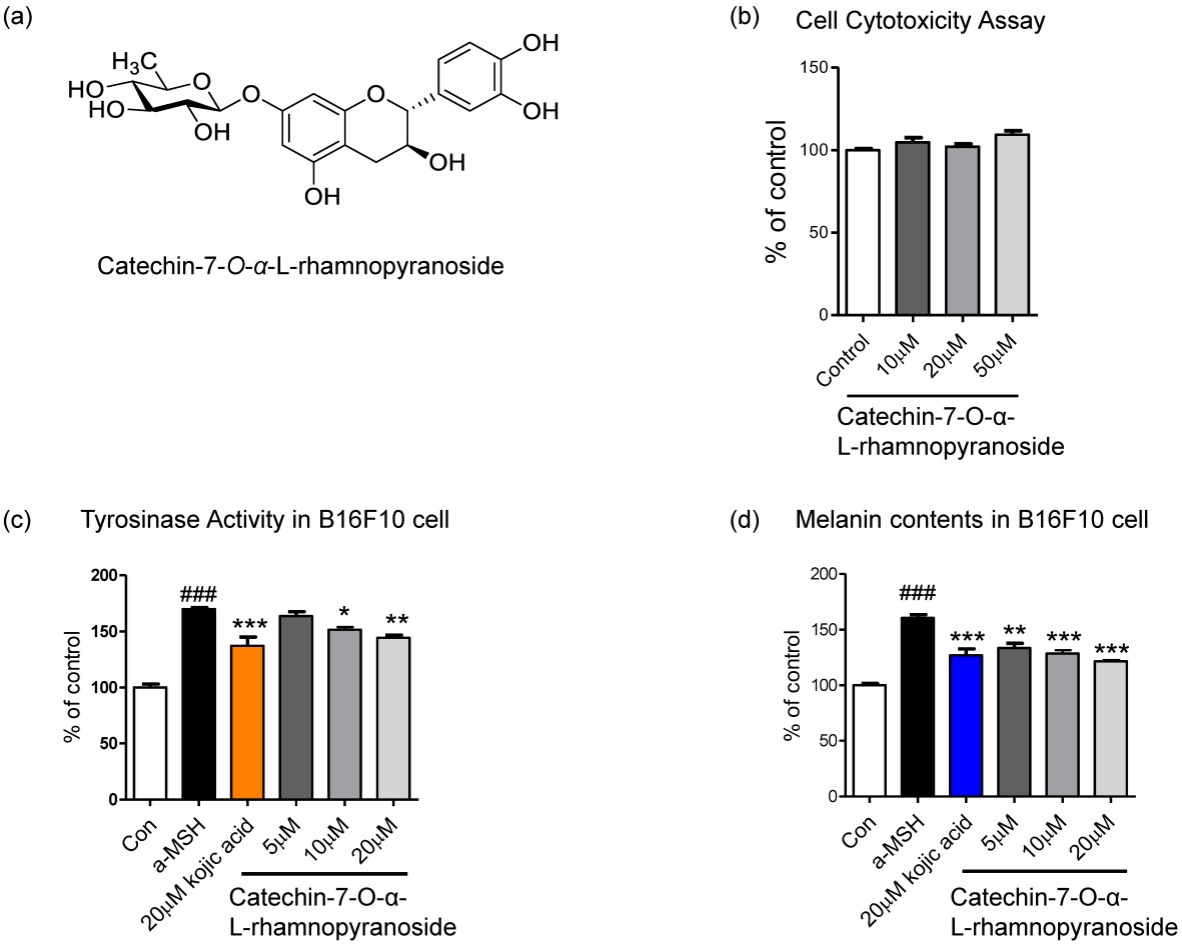
** Inhibitory effect of C7R on melanogenesis in B16F10 melanoma cells. (A)** The chemical structure of C7R. **(B)** Cell viability assay was tested in B16F10 melanoma cells with various doses (10-50 µM) of C7R for 72 hours. The cell viability was measured by cell cytotoxicity assay solution (See based on materials and methods). The results are expressed as the relative percentage of the untreated group (n=3/group). B16F10 melanoma cells were pretreated with C7R or kojic acid for 3 hours, and then the cells were stimulated with 1 µM α-MSH for **(C)** 72 hours or **(D)** 48 hours at each concentration, respectively. All data (n=3) represent the mean ± SEM. Data were analyzed using one-way ANOVA and then Dunnett's test. *^###^p* < 0.001 compared to non-treated group. ^*^p < 0.05, ^**^p < 0.01, ^***^p < 0.001 compared to the α-MSH treated group.

C7R is a catechin glycoside that belongs to phenols and flavonoids. It has a flavan-3-ol backbone and a rhamnopyranose moiety at the 7^th^ carbon (Fig. 1a). To confirm that C7R is not cytotoxicity, we performed a cell viability assay. And we then tested whether an inhibitory effect of C7R on melanin biosynthesis and tyrosinase activity in B16F10 cells. This compound showed no cytotoxicity at 10-50 μM in B16F10 cells (Fig. 1b). The inhibitory melanogenesis effect of C7R was measured at non-cytotoxic concentrations with kojic acid as a positive control. Cells were pretreated with various concentrations of C7R (5-20 μM) for 24 hours as well as 20 μM kojic acid for 24 hours. Melanogenesis and tyrosinase were induced by α-MSH for 2 days or 3 days. As shown in the Figure 1c-d, the results demonstrated that C7R notably decreased α-MSH-induced melanin contents and tyrosinase activity in B16F10 melanoma cells in a dose-dependent manner. Furthermore, the brightening effects of C7R in cell culture media were also observed (Fig. 1). In addition, when C7R was treated with the same concentration as kojic acid, a well-known positive control, it showed a similar melanogenesis and inhibition of tyrosinase activity compared to kojic acid (Fig. 1c-d). From these things, it was suggested that C7R has an anti-melanin effect on α-MSH-induced melanogenesis.

### Inhibitory effect of C7R on tyrosinase activity *in vitro*

**Figure 2:**
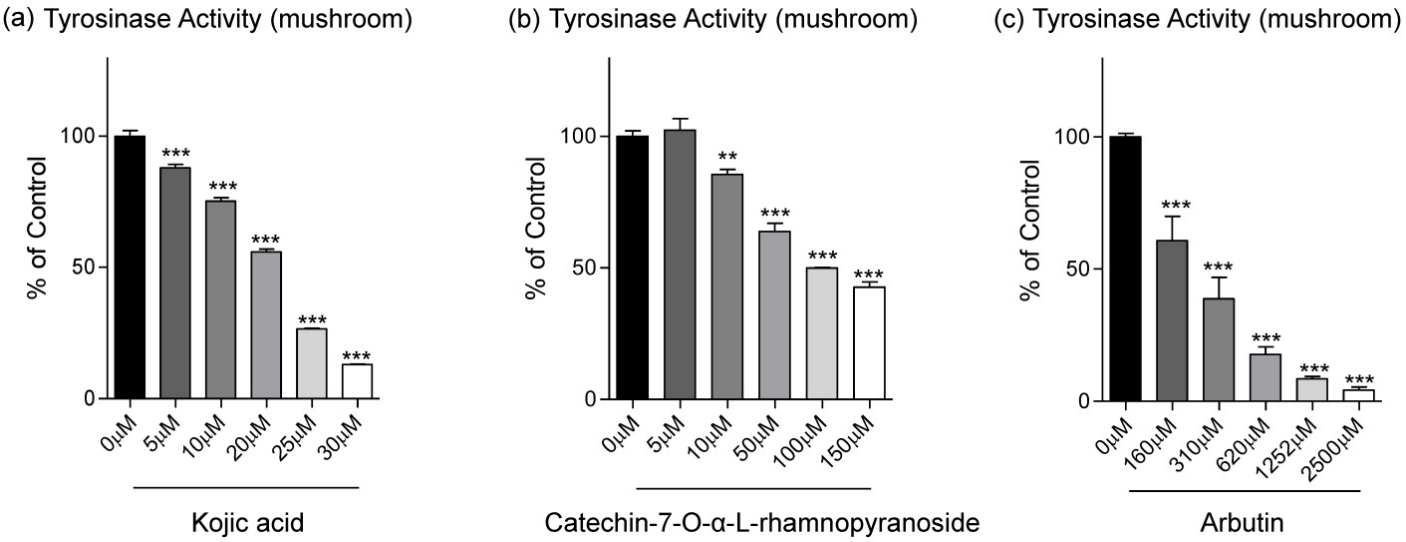
** Inhibitory effects of C7R on tyrosinase activity in the cell-free system. (A)** Kojic acid, **(B)** C7R, and **(C)** arbutin were used to determine the effect on tyrosinase activity by using 1 mM L-tyrosine as the substrate and mushroom tyrosinase (500 units) in various concentrations, respectively. The results are expressed as the relative percentage of the untreated group. All data (n=3) represent the mean value ± SEM. Data were analyzed using one-way ANOVA and then Dunnett's test. ^*^p < 0.05, ^**^p < 0.01, ^***^p < 0.001 compared to the untreated group (n=3/group).

From data in Fig. 1d, C7R has an inhibitory effect on tyrosinase activity in B16F10 cells. To evaluate on direct inhibition of tyrosinase activity of C7R, we further analyzed the dose-dependent inhibitory effect of C7R on tyrosinase activity, and IC_50_ values were calculated (Table 1). From Table 1, in our *in vitro* experiment using tyrosinase purified from a mushroom, the IC_50_ value of C7R was 113.1 μM while IC_50_ values of kojic acid and arbutin as positive control were 18.4 μM and 306.1 μM, respectively. Although the IC_50_ value of kojic acid indicates a better inhibitory effect on tyrosinase activity than C7R, it is a new compound for a tyrosinase inhibitor, at least, much better than arbutin (Fig. 2).

### The *in vitro* mechanism on the inhibition of tyrosinase activity by C7R

**Figure 3:**
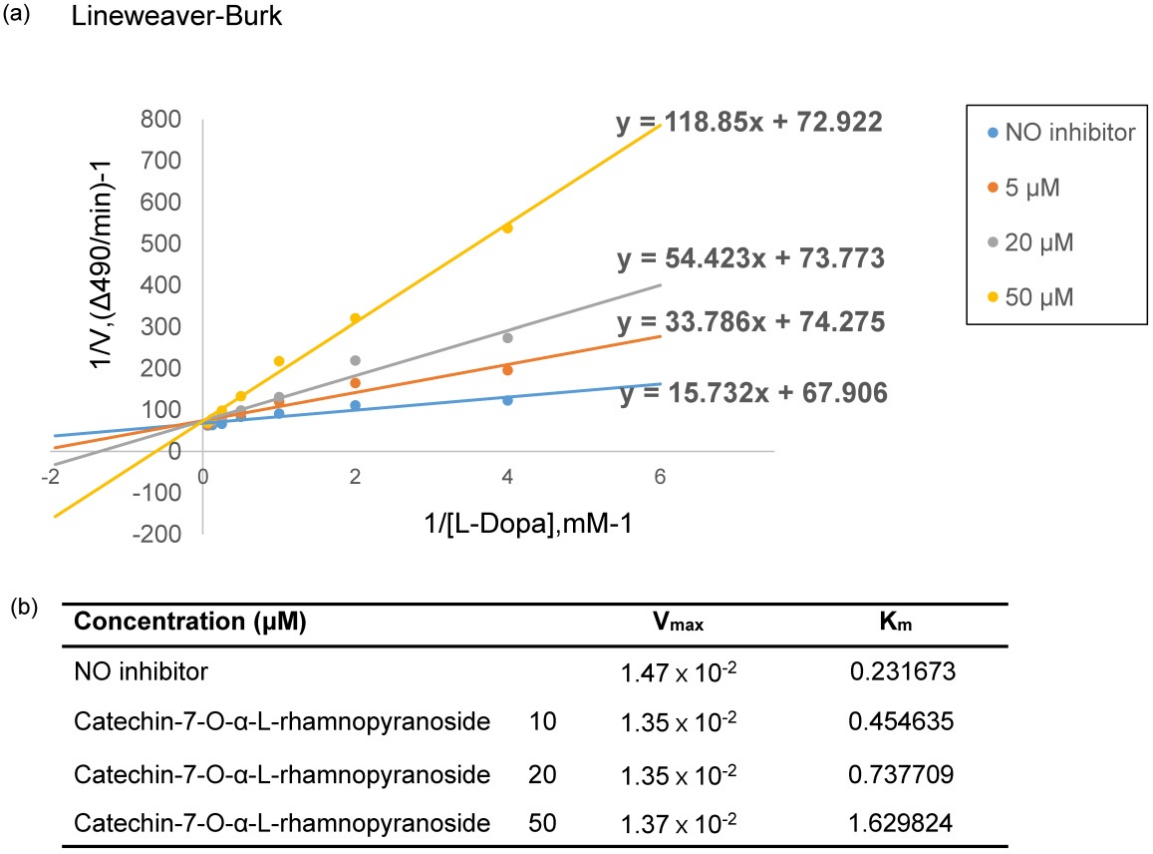
** C7R competitively inhibits tyrosinase. (A)** Lineweaver-Burk plot and kinetic analysis were performed to investigate the inhibition mode by C7R on mushroom tyrosinase. The inhibitory tyrosinase mechanism of C7R was investigated using a kinetic assay with various concentrations of L-DOPA (0.45, 0.73, 1.62 mM) and C7R (0, 10, 20, and 50 µM). The Lineweaver-Burk plot was made by calculating the reciprocal of each value. On the basis of the point of convergence of lines on the plot that shows the inverse of reaction velocity (1/*v*) vs. the inverse of substrate concentration (1/[S]), an inhibitory mechanism was determined. **(B)** *V*_max_ is the maximum reaction rate based on the Line Weaver-Buck plot result, and *K*_m_ is the Michaelis-Menten constant.

To elucidate the mechanism of inhibition on tyrosinase activity by C7R, the Lineweaver-Burk analysis was performed. From Fig. 3a, four lines of different concentrations of C7R intersected at the same y-axis. These data suggest that C7R inhibits tyrosinase activity in a competitive manner. Furthermore, when C7R was applied with various concentrations of 10, 20, 50 μM, respectively, resulting Michalis constants (*K*_m_) were 0.45, 0.73, 1.62 mM and maximum velocity (*V*_max_) was 1.35×10^-2^ mM/min (Fig. 3b). Because *K*_m_ for tyrosinase increased in a dose-dependent manner without changing *V*_max_, these results demonstrate a competitive inhibition of tyrosinase by C7R. And these results were consistent with those of kojic acid, which is a known competitive inhibitor of tyrosinase 17.

### Binding of C7R to tyrosinase by using *in silico* analysis

**Figure 4:**
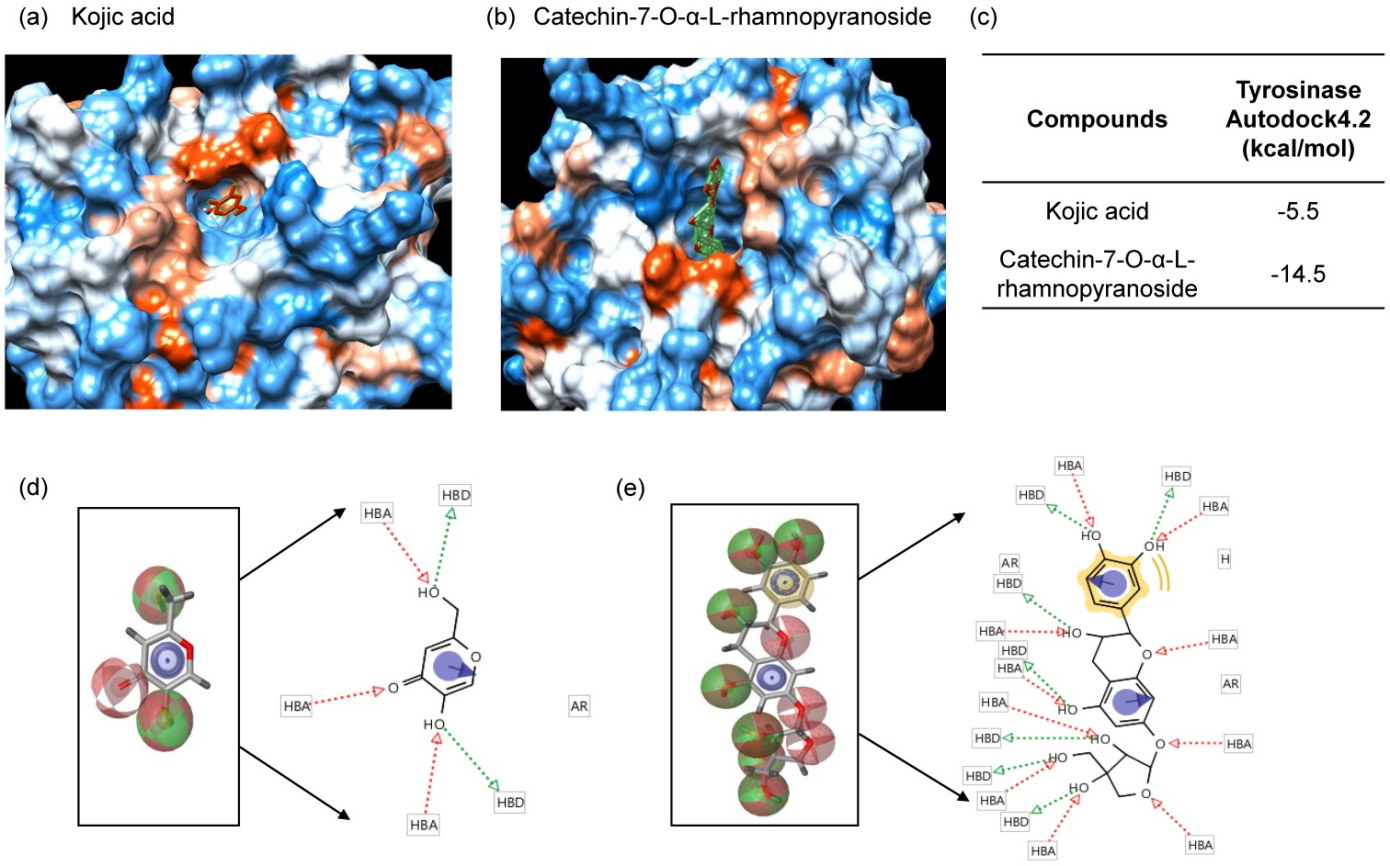
***In silico* docking simulation and pharmacophore analysis of tyrosinase and C7R.** Protein-ligand docking simulation was analyzed using AutoDock 4.2. Docking simulation is the result between the expected 3D structure of tyrosinase, and **(A)** kojic acid and **(B)** C7R, respectively. As the 3D structure of tyrosinase molecule, we used the crystal structure of Agaricus bisporus (PDB ID: 2Y9X). **(C)** The binding energy values of kojic acid and C7R were -5.5 and -14.5 kcal/mol, respectively. It was the binding residues analyzed between human tyrosinase and **(D)** kojic acid, and **(E)** C7R as a pharmacophore analysis.

Since C7R inhibited tyrosinase activity *in vitro* analysis using a mushroom tyrosinase, it is expected that C7R directly binds to tyrosinase and then inactivates tyrosinase. To confirm this idea, *in silico* docking simulation was conducted using the AutoDock 4.2 program. The expected binding energy affinity values between mushroom tyrosinase (PDB ID: 2Y9X) and C7R were investigated in three dimensional (3D) structures. From Fig. 4, the docking simulation image showed that C7R binds to the known active site of tyrosinase. When C7R was combined with the active site pocket of tyrosinase, the binding energy value was -5.5 kcal/mol for kojic acid, whereas the value for C7R was -14.5 kcal/mol (Fig. 4a-c). These results indicated that C7R may bind to tyrosinase active site as well as it was much higher than kojic acid for inhibitory tyrosinase activity.

For a pharmacophore analysis, we used Ligand Scout 3.1 program to investigate amino acid residues of tyrosinase that bind to C7R or kojic acid. Kojic acid can have three hydrogen-bonded receptors and two hydrogen-bonded donors associated with tyrosinase binding, and also can form one aromatic interaction with tyrosinase. On the other hand, C7R can have ten hydrogen-bonded receptors and seven hydrogen-bonded donors associated with tyrosinase binding, and also can form one hydrophobic and one aromatic interaction with tyrosinase (Fig. 4d-e).

## Discussion

This study was conducted to discover a new natural compound with whitening effects like kojic acid and arbutin which reduce melanogenesis by inhibiting tyrosinase activity in B16F10 cells. Here, we investigated the effect of C7R on α-MSH-induced melanogenesis in B16F10 cells. In addition, we also assessed anti-melanogenesis effects of C7R through *in vitro* and *in silico* analysis. C7R did not cause cytotoxicity in B16F10 melanoma cells, and the pretreatment with C7R suppressed α-MSH induced-melanogenesis and tyrosinase activity in B16F10 melanoma cells. While C7R has a lower effect on inhibition of purified mushroom tyrosinase compared to kojic acid, it inactivates tyrosinase in B16F10 melanoma cells as much as kojic acid. From our docking simulation and pharmacophore analysis, C7R may inhibit tyrosinase activity by directly binding to tyrosinase because of forming multiple hydrogen bonds, aromatic and hydrophobic interactions with the active site of tyrosinase. Together, these results indicate that C7R can reduce melanogenesis by inhibiting tyrosinase.

Interestingly, it was reported that the catechin family has a skin whitening effect by suppressing tyrosinase expression 16. In addition, it was suggested that the catechin structure is a candidate for the anti-melanogenesis agent and may be effective in hyperpigmentation disorders. In this study, we showed that C7R has inhibitory effects on tyrosinase activity. However, it may also affect tyrosinase expression and this possibility should be tested in further study.

Currently, some whitening ingredients are used in clinical practice, but side effects of them are still problematic. For example, kojic acid is an effective compound that inhibits melanogenesis and has been used continuously in cosmetics. But its use was banned in 2003 due to safety problems 10. On the other hand, in November 2005, the Japanese Ministry of Health, Labor, and Welfare conducted a toxicity test of kojic acid and confirmed that there was no particular problem in safety. And although the use of kojic acid in cosmetics is now permitted, it is still controversial for its use 18, 19. In addition, kojic acid is not oil-soluble and is unstable at high temperature when stored for a long period of time. Thus, because of these disadvantages, it cannot be added to cosmetic products that are oil-soluble as raw materials 12, 18. For another example, arbutin which is another whitening ingredient has a structure in which glucose is bound to hydroquinone. Thus it is likely to be decomposed by high temperature and UV 11. Furthermore, there are safety issues such as skin toxicity and skin inflammation because it can produce hydroquinone, which is known for causing skin disease and cancer 11. Whereas, a natural product C7R is potentially safer than kojic acid and arbutin from the cell experimental results, suggesting its potential use as an excellent whitening agent. However, additional experiments are required to reconfirm the melanin inhibitory effect and safety of C7R using artificial skin or in an animal model if possible.

In conclusion, C7R efficiently inhibited α-MSH-induced melanogenesis in B16F10 melanoma cells without cytotoxicity. In addition, C7R shows a competitive inhibition of tyrosinase activity like kojic acid and arbutin. Our results also indicate that C7R directly binds to tyrosinase and competitively inhibits tyrosinase in melanocytes. Thus, our results suggest that C7R can be applied as a novel modulating molecule to control skin aging process and/or pigmentation disorders.

## Materials and Methods

### Materials

Tyrosinase from mushroom (T3824-25KU, tyrosinase activity 7164 unit/mg solid), L-3,4-dihydroxyphenlyalanine (L-DOPA, D9628), 3-(4-Hydroxyphenyl)-L-alanine (L-tyrosine, T3754), 5-hydroxy-2-(hydroxymethyl)-4H-pyran (kojic-acid), Arbutin and α-Melanocyte Stimulating Hormone (α-MSH, M4135) were purchased from Sigma Aldrich (St, Louis, US). Dulbecco modified Eagle medium (DMEM), Fetal Bovine Serum (FBS), Phosphate Buffered Saline (PBS), and Trypsin were purchased from WELGENE (Gyeongsangbuk-do, Korea). Cell Viability Assay Kit (EZ-Cytox) was purchased from DOGEN (Seoul, Korea).

### Sample preparations

C7R was isolated from the stem bark of *U. parvifolia* in our laboratory according to our previously reported method 20. The chemical structure was identified by comparing the MS, ^1^H- and ^13^C-NMR spectroscopic data with the previously reported ones 21.

### Cell culture and cell cytotoxicity assay

B16F10 mouse melanoma cells were purchased from the Korean cell line bank (Seoul, Korea). B16F10 melanoma cells were cultured in DMEM (Dulbecco's Modified Eagle Medium; WELGEN) medium at 37 °C including 2 mM L-glutamine, 100 units/mL streptomycin, and 10% heat-activated FBS (fetal bovine serum; WELGENE). Cells were incubated at 37°C in a humidified atmosphere with 5% CO_2_.

To measure cell cytotoxicity with an EZ-Cytox assay kit, B16F10 melanoma cells (1ⅹ10⁴ cells/well) were seeded in 96 well plates for 24 hours (e.g., at 37 °C, 5% CO_2_). B16F10 melanoma cells were pretreated with 10-50 μM of the C7R to each well. After 72 hours, each well was added to 10 μL of EZ-Cytox and reacted in the incubator for 2 hours, and then shaken for 1 minute before measuring the absorbance. The absorbance was measured at 450 nm using a plate reader.

### Measurement of melanin contents in B16F10 cells

To investigate melanin contents, cells were pretreated with C7R at various concentrations (5-20 µM) for 1hr, and then melanogenesis was stimulated by 1 μM α-MSH for 3 days at each concentration. Cells lysis was used with 1 N sodium hydroxide (NaOH). Melanin contents were measured of absorbance at 490 nm using a microplate reader, and as a positive control, kojic acid. The level of melanin was expressed as a percentage compared to the untreated α-MSH.

### Tyrosinase activity assay *in vitro*

Tyrosinase activity was measured using tyrosinase purified from a mushroom as previously described method 6. In cell free-system, 20 μL of 1 mM L-tyrosine substrate was used in 170 μL of 50 mM sodium phosphate buffer (pH 6.8) and 10 μL of C7R (5, 50, 100, 150, 250 μM) or positive control, and then 20 μL of mushroom tyrosinase (500 units) were added to each well and incubated at 37 ˚C for 30 min. The generated DOPA chrome was measured at 475 nm wavelength. Tyrosinase inhibitory activity was expressed as the absorbance reduction rate of the group with and without the sample solution. Based on the measurement of C7R on tyrosinase activity, tyrosinase inhibitory concentration 50 (IC_50_) was calculated using a log-linear curve and its equation.

To evaluate the tyrosinase inhibitory mechanism of C7R, a kinetic assay was used with various concentrations of L-DOPA (0.125-4 mM) and C7R. And kojic acid and arbutin were also used as positive controls. The values of the kinetic constants were calculated using Lineweaver-Burk plot analysis, and the maximum velocity (*V*_max_) and Michaelis constant (*K*_m_) were also calculated by the Lineweaver-Burk plots with the various concentration of L-DOPA substrate 22.

### *In silico* docking simulation between C7R to tyrosinase

AutoDock 4.2 was used to perform *in silico* protein-ligand docking simulations. In order to obtain a successful binding between the protein and the ligand, the crystal structure from Agaricus bisporus (PDB ID: 2Y9X) as the 3D structure of tyrosinase was used, and then a predefined tyrosine binding site was designated and applied as a docking pocket. After performing docking simulations between tyrosinase and C7R or kojic acid, the pharmacophores analysis was used by Ligand Scout 3.0 to find the prediction of possible binding residues between compounds and tyrosinase.

### Statistical analysis

The experimental group in each experiment was analyzed by one-way ANOVA using the Dunnette test. *P-values* less than 0.05 were reported as significant. Data were presented as means ± SEM of at least three independent experiments conducted in triplicate.

## Figures and Tables

**Table 1:** IC_50_ (µM) values for Catechin-7-O-α-L-rhamnopyranoside

Compounds	Concentration (μM)	Tyrosinase Inhibition (%)	IC_50_ (μM)
Kojic acid	0	100 ± 3.74	18.40
	5	87.92 ± 2.30	
	10	75.28± 2.25	
	20	55.87 ± 1.79	
	25	26.52± 0.39	
Catechin-7-O-α-L-rhamnopyranoside	0	100 ± 3.65	113.05
	5	102.33 ± 7.60	
	50	85.52± 3.42	
	100	63.85 ± 5.46	
	150	49.79± 0.41	
	250	42.60± 3.48	
Arbutin	0	100 ± 2.39	306.09
	160	60.73 ± 15.96	
	310	38.71 ± 14.12	
	620	17.64 ± 5.05	

## References

[1] Videira IF, Moura DF, Magina S (2013). Mechanisms regulating melanogenesis. An Bras Dermatol.

[2] Briganti S, Camera E, Picardo M (2003). Chemical and instrumental approaches to treat hyperpigmentation. Pigment Cell Res.

[3] Ni-Komatsu L, Tong C, Chen G, Brindzei N, Orlow SJ (2008). Identification of quinolines that inhibit melanogenesis by altering tyrosinase family trafficking. Mol Pharmacol.

[4] Jeong CH, Shim KH (2004). Tyrosinase inhibitor isolated from the leaves of Zanthoxylum piperitum. Biosci Biotechnol Biochem.

[5] Nihei K, Yamagiwa Y, Kamikawa T, Kubo I (2004). 2-hydroxy-4-isopropylbenzaldehyde, a potent partial tyrosinase inhibitor. Bioorg Med Chem Lett.

[6] Lee B, Moon KM, Lim JS, Park Y, Kim DH, Son S (2017). 2-(3, 4-dihydroxybenzylidene)malononitrile as a novel anti-melanogenic compound. Oncotarget.

[7] Lee B, Moon KM, Kim SJ, Kim SH, Kim DH, An HJ (2016). (Z)-5-(2,4-Dihydroxybenzylidene)thiazolidine-2,4-dione Prevents UVB-Induced Melanogenesis and Wrinkle Formation through Suppressing Oxidative Stress in HRM-2 Hairless Mice. Oxid Med Cell Longev.

[8] Moon KM, Jeong JW, Lee B, Kim DH, Kim HR, Woo YW (2016). Antimelanogenic activity of MHY384 via inhibition of NO-induced cGMP signalling. Exp Dermatol.

[9] Sturm RA, Box NF, Ramsay M (1998). Human pigmentation genetics: the difference is only skin deep. Bioessays.

[10] Morison WL (1985). What is the function of melanin?. Arch Dermatol.

[11] Hincha DK, Oliver AE, Crowe JH (1999). Lipid composition determines the effects of arbutin on the stability of membranes. Biophys J.

[12] Kvam E, Tyrrell RM (1999). The role of melanin in the induction of oxidative DNA base damage by ultraviolet A irradiation of DNA or melanoma cells. J Invest Dermatol.

[13] Song ARBH (2006). eFloras: New directions for online floras exemplified by the Flora of China Project. Flora of China.

[14] Mina SA, Melek FR, Adeeb RM, Hagag EG (2016). LC/ESI-MS/MS profiling of Ulmus parvifolia extracts and evaluation of its anti-inflammatory, cytotoxic, and antioxidant activities. Z Naturforsch C J Biosci.

[15] Kang MC, Yumnam S, Park WS, So HM, Kim KH, Shin MC (2019). Ulmus parvifolia Accelerates Skin Wound Healing by Regulating the Expression of MMPs and TGF-beta. J Clin Med.

[16] Sato K, Toriyama M (2009). Depigmenting effect of catechins. Molecules.

[17] Cardoso R, Valente R, Souza da Costa CH, da SGVJL Jr, Santana da Costa K, de Molfetta FA (2021). Analysis of Kojic Acid Derivatives as Competitive Inhibitors of Tyrosinase: A Molecular Modeling Approach. Molecules.

[18] Burnett CL, Bergfeld WF, Belsito DV, Hill RA, Klaassen CD, Liebler DC (2010). Final report of the safety assessment of Kojic acid as used in cosmetics. Int J Toxicol.

[19] Cheng SL, Huang Liu R, Sheu JN, Chen ST, Sinchaikul S, Tsay GJ (2006). Toxicogenomics of kojic acid on gene expression profiling of a375 human malignant melanoma cells. Biol Pharm Bull.

[20] Park WS, Kim HJ, Khalil AAK, Kang DM, Akter KM, Kwon JM (2021). Anatomical and Chemical Characterization of Ulmus Species from South Korea. Plants (Basel).

[21] Inoshiri S SM, Kohda H, Otsuka H, Yamasaki K (1987). Aromatic glycosides from Berchemia racemosa. Phytochemistry.

[22] Lee B, Moon KM, Lee BS, Yang JH, Park KI, Cho WK (2017). Swertiajaponin inhibits skin pigmentation by dual mechanisms to suppress tyrosinase. Oncotarget.

